# Strychnine in Chorea

**Published:** 1850-03

**Authors:** 


Strychnine in Chorea.—M. Trousseau treats chorea by the use of a preparation of the sulphate of strychnine. Of this, they prepare a syrup in the proportion of four-fifths of a grain of strychnine to three ounces of simple syrup. In children from six to twelve years of age, he commences with six teaspoonfuls during the day; in more advanced age, the dose is a desertspoonfull six times a day. The doses are equivalent in the first case to thirty, and in the second to fifty grammes of the syrup, twenty grammes of which contain one-seventh of a grain of strychnine. These doses were increased or diminished according to the effects produced. Under the use of the remedy, a distinct rigidity of the jaws, neck, and limbs is produced; but the author has found that these physiological symptoms are the forerunners of the yielding of the disease, and they therefore advise the continuance of the medicine until they are induced.—*Ranking's Abstract.*

